# The effects of maternal fish oil supplementation rich in n-3 PUFA on offspring-broiler growth performance, body composition and bone microstructure

**DOI:** 10.1371/journal.pone.0273025

**Published:** 2022-08-16

**Authors:** Yuguo H. Tompkins, Chongxiao Chen, Kelly M. Sweeney, Minjeong Kim, Brynn H. Voy, Jeanna L. Wilson, Woo Kyun Kim

**Affiliations:** 1 Department of Poultry Science, University of Georgia, Athens, Georgia, United States of America; 2 Department of Animal Science, University of Tennessee, Knoxville, Tennessee, United States of America; Tokat Gaziosmanpasa Universitesi, TURKEY

## Abstract

This study evaluated the effects of maternal fish oil supplementation rich in n-3 PUFA on the performance and bone health of offspring broilers at embryonic development stage and at market age. Ross 708 broiler breeder hens were fed standard diets containing either 2.3% soybean oil (SO) or fish oil (FO) for 28 days. Their fertilized eggs were collected and hatched. For a pre-hatch study, left tibia samples were collected at 18 days of incubation. For a post-hatch study, a total of 240 male chicks from each maternal treatment were randomly selected and assigned to 12 floor pens and provided with the same broiler diets. At 42 days of age, growth performance, body composition, bone microstructure, and expression of key bone marrow osteogenic and adipogenic genes were evaluated. One-way ANOVA was performed, and means were compared by student’s t-test. Maternal use of FO in breeder hen diet increased bone mineral content (*p* < 0.01), bone tissue volume (*p* < 0.05), and bone surface area (*p* < 0.05), but decreased total porosity volume (*p* < 0.01) during the embryonic development period. The FO group showed higher body weight gain and feed intake at the finisher stage than the SO group. Body composition analyses by dual-energy X-ray absorptiometry showed that the FO group had higher fat percentage and higher fat mass at day 1, but higher lean mass and total body mass at market age. The decreased expression of key adipogenic genes in the FO group suggested that prenatal FO supplementation in breeder hen diet suppressed adipogenesis in offspring bone marrow. Furthermore, no major differences were observed in expression of osteogenesis marker genes, microstructure change in trabecular bone, or bone mineral density. However, a significant higher close pores/open pores ratio suggested an improvement on bone health of the FO group. Thus, this study indicates that maternal fish oil diet rich in n-3 PUFA could have a favorable impact on fat mass and skeletal integrity in broiler offspring.

## Introduction

With the interest in good quality and high meat yield, the growth rate of broilers has been significantly improved to meet the product demand in the modern poultry industry [1]. Due to inadvertent consequences of selection for rapid growth, the body fat portion of broiler chickens has decreased [2], however, the genetic tendency for broilers to accumulate more adipose tissue that is physiologically necessary has increased [2, 3]. Moreover, the selection programs pose many challenges particularly those related to bone health. Broiler skeletal abnormalities, including rickets and bacterial chondronecrosis with osteomyelitis caused lameness, elevated not only production costs, but also the public’s concern for animal welfare [4–6]. Thus, it suggests that progress should be made in improving bone health in the modern broilers.

In recent years, the health benefits of polyunsaturated fatty acids, the long-chain omega-3 polyunsaturated fatty acids (LC n-3 PUFA) in particular, have drawn a lot of attention from both the public and researchers. Metabolic benefits of LC n-3 PUFA are commonly attributed to eicosapentaenoic acid (EPA; 20–5 n-3) and docosahexaenoic acid (DHA; 22:6 n-3) [7]. Fish oil is not only the best resource of DHA and EPA, but also one of the most extensively researched nutritional supplements. It benefits a variety of metabolic aspects including development and growth [8–11]. Dietary lipids play an important role in the development and remodeling of long bones in broiler chicks [12, 13]. Several studies have suggested the positive effects of dietary fish oil on bone health in the broiler production [14–16]. Bone content of EPA and DHA, which can be enriched by the diet, has been shown to correlate directly with bone mineral content, bone density, and resistance to force in rat [17–19]. Compared to vegetable oil, dietary fish oil supplementation increased cortical thickness and bone ash content in Japanese quail [18, 20]. Dietary LC n-3 PUFA can exert additional benefits for bone strength through their effects on development and metabolic activities of osteoblasts and osteoclasts, shown to promote osteoblastogenesis and suppress the formation of osteoclasts when comparable to LC n-6 PUFA [21–23]. Other than promoting bone quality, n-3 PUFA also decrease adipose tissue in part due to the shared developmental origin of adipocytes and osteoblasts, both of which originate during embryonic development from mesenchymal stem cells [24]. Dietary EPA and DHA supplementation suppressed the differentiation and maturation of adipocytes, resulting in lower fat accretion in humans, mammals, and broilers [25–28].

Studies indicate that energy metabolism and adiposity are especially sensitive to developmental programming by the maternal diet in mammals and avian species [29–31]. Developmental programming refers to the embryonic environment encounter of persistent effects that affected the physiology, metabolism and epigenome of offspring after birth [32, 33]. Developmental programming is a particularly attractive tool for use in broiler production because manipulations would be applied at the level of the broiler breeder hen or the *in ovo* environment. Maternal consumption of fish oil is well-documented in rodents and humans but limited in avian species [29–31, 34–36]. The hen diet is a practical way to manipulate the embryonic fatty acids profile, because fatty acids are required for chick embryo development, and fatty acid profile of the yolk could be affected by hen diet [37, 38]. Thus, fish oil supplementation in breeder hen diet potentially alleviates excess fat deposition and bone weakness in offspring broilers [3]. Work by Liu et al. [18] using quail supports the potential to developmentally program bone quality through fish oil in the hen diet, and extensive evidence from several studies in other species indicated the maternal dietary EPA and DHA are not only associated with lower fat accretion but also better bone quality in offspring [14, 25–28, 39–42]. Maternal supplementation of fish oil in broiler breeder hens decreased adiposity in offspring broiler chicks compared to those hatched from hens fed fatty acids from corn oil (an n-6 PUFA-rich oil) [43]. With conservation of osteogenic development between avian and other mammal species, it is reasonable to hypothesize that developmental programming with LC n-3 PUFA may have similar benefits for bone quality. The objective of the current study was to evaluate the effect of maternal fish oil on offspring broilers body composition and bone quality. The understanding of the interaction between prenatal nutrition and offspring growth may provide a new insight for avian bone development and novel nutritional means to enhance bone health in broilers.

## Materials and methods

### Ethics statement

All experiments followed the guidelines of the Institutional Animal Care and Use Committee and was conducted at the Poultry Research Farm, University of Georgia, Athens, GA. The protocol was approved by the Institutional Animal Care and Use Committee at the University of Georgia.

### Experimental design

Ross 708 broiler breeder hens (N = 40/diet) were fed standard diets containing 2.3% of either soybean oil (SO; Conagra Brands; Chicago, IL) or fish oil (FO; Jedwards International, Braintree MA) for 28 days. The FO contained 18% EPA and 12% DHA. Management and diet formulation were as previously described [43]. Fertilized eggs were collected over a period of two weeks and placed in an egg cooler that was held between 65–68°F and 55–65% relative humidity. Later eggs were incubated under standard conditions with 99.5°F and 60% relative humidity. Chicks from the SO-fed hens were named the SO group, and chicks from the FO-fed hens were named the FO group. For a pre-hatch bone development study, 10 embryos from each group were randomly chosen at embryonic day 18 and euthanized by cervical dislocation. For a post-hatch study, a total of 120 one-day-old male chicks from each treatment were randomly selected and distributed to 6 floor pens (240 chicks in total, six replicate floor pens per treatment with 20 birds per pen). Each pen was equipped with a hanging feeder, a nipple drinker line, and fresh wood shavings litter. All chicks were raised to 42 days of age in the same room. Feeding and environmental management conditions were based on broiler recommendations for the Ross 708 [44]. Chicks were allowed to consume feed and water on an *ad libitum* basis. In each experiment, all chicks were fed the same corn-based diet after hatch, ensuring the changes in body composition were due to the maternal hen diet. A three phase feeding program with starter (1–14 days of age), grower (15–28 days of age), and finisher (29–42 days of age) diets in mash form were used based on the nutritional requirements of Ross broilers (Table 1) [45].

**Table 1:** Composition and calculated contents of the experimental diets.

**Item**	**0–14 d**	**15–28 d**	**29–42 d**
Ingredients, %			
Corn, Grain	62.85	67.34	68.84
Soybean meal -48%	33.32	28.52	26.36
Dicalcium Phosphate	1.80	1.66	1.46
Corn oil	0.41	0.93	1.93
Limestone	0.52	0.51	0.50
Common salt	0.23	0.21	0.20
DL-methionine	0.28	0.24	0.20
Vitamin Premix^1^	0.25	0.25	0.25
L-lysine-HCL	0.17	0.16	0.11
Threonine	0.05	0.04	0.03
Mineral premix^2^	0.08	0.08	0.08
Antiprotozoal agent^3^	0.05	0.05	0.05
**Energy and nutrient composition**			
ME, kcal/kg	3008	3086	3167
Crude protein %	21.00	19.00	18.00
Lysine %	1.18	1.05	0.95
Methionine %	0.45	0.42	0.39
Arginine %	1.24	1.10	1.03
Threonine %	0.77	0.69	0.65
Valine %	0.89	0.81	0.73
Tryptophan %	0.18	0.17	0.17
Total sulfur amino acid %	0.88	0.80	0.74
Ca %	0.90	0.84	0.76
Available P %	0.45	0.42	0.38

^1^Vitamin premix include provides the following per kg of diet: Vitamin A 2,204,586 IU, Vitamin D3 200,000 ICU, Vitamin E 2,000 IU, Vitamin B12 2 mg, Biotin 20 mg, Menadione 200 mg, Thiamine 400 mg, Riboflavin 800 mg, d-Pantothenic Acid 2,000 mg, Vitamine B6 400 mg, Niacin 8,000 mg, Folic Acid 100 mg, Choline 34,720 mg.

^2^Mineral premix provides the following per kg of diet: Ca 0.72 g, Mn 3.04 g, Zn 2.43 g, Mg 0.61 g, Fe 0.59 g, Cu 22.68 g, I 22.68 g, Se 9.07 g.

^3^ Coban-90 (Elanco Animal Health, Indianapolis, IN): Monensin was included at 99 mg per kg of diet.

### Growth performance

Body weight (BW) and feed intake (FI) per pen were recorded at 1, 14, 28, and 42 days of growth. The body weight gain (BWG) and feed conversion ratio were calculated in each feeding phase and overall period. Birds were monitored more than twice a day, and any mortalities were weighted to adjust feed conversion ratio.

### Body composition

Dual energy x-ray absorptiometry (DEXA; GE Healthcare, Chicago, IL) was used to determine the effect of maternal fish oil intake on body composition in offspring broilers. Three birds per pen were randomly selected for body composition measurement at day 1 and day 42. After euthanasia, the birds were placed face-up on the DEXA scanner and scanned using a small animal software module (Lunar Prodigy from GE, encore software version 12.20.023). Defining the whole bird as a region of interest (ROI), the DEXA provided measurements in bone mineral content (BMC), bone mineral density (BMD), fat mass, lean mass, fat percentage, lean percentage, and total tissue mass for each bird.

### Micro-computed tomography (μCT)

For the pre-hatch study, left tibias were collected from day 18 embryos. Microstructure of tibia metaphysis section was measured by Micro-Computed Tomography (μCT) according to a standard protocol at 82 kV, 121 μA, and a 0.5 mm aluminum filter, and analyses were performed with a SkyScan 1172 (SkyScan, Kontich, Belgium) [46, 47]. For the post-hatch study to evaluate bone morphologic changes in the broiler, 36 samples (18 samples per treatment group) were randomly chosen at day 42. In order to fit the test space and specifications, the right femurs were scanned at 75 kV, 126 μA, and a 0.5 mm aluminum filter. The pixel size was fixed at 26 μm, and a 0.25° rotation angle was applied at each step. 2-D images were transferred to CTAn software (CTAn, SkyScan) for structure construction and quantification. Cortical bone and trabecular bone structures were reconstructed respectively by CTAn software and separated for various bone parameter analyses. The following parameters were quantified: Tissue Volume (TV), Bone Volume (BV), Bone Volume per Tissue Volume (BV/ TV), Trabecular Number (Tb. N), Trabecular Thickness (Tb. Th), Trabecular Separation (Tb. Sp), Connectivity Density, Structure Model Index (SMI), Total surface area (TS), Bone surface area (BS), Total Porosity (Po (tot)), Volume of Pores (Po. V (tot)), Open Pore Percentage (Po. (op)), Close Pore Percentage (Po. (cl)), Number of Closed Pores (Po. N (cl)), Number of Open Pores (Po. N (op)), Volume of Open Pores (Po. V (op)) and Closed Pores Surface (Po. S(cl)).

### Real-time quantitative PCR analysis of gene expression in bone marrow

Left femurs were collected at day 42. After bones were opened, whole bone marrow was extracted and stored immediately at -80°C until RNA isolation (n = 6). Total RNA from bone marrow was extracted using Qiazol reagents (Qiagen, USA) according to the manufacturer’s instructions. A Nano-Drop 1000 Spectrophotometer (ThermoFisher Scientific, Pittsburgh, PA) was used to determine the quantity of extracted RNA. The cDNA was synthesized from total RNA (2,000 ng) using high-capacity cDNA reverse transcription kits (Thermo Fisher Scientific, Waltham, MA).

Quantitative real-time reverse transcription polymerase chain reaction (qRT-PCR) was used to measure mRNA expression. Primers were designed using the Primer-BLAST program (https://www.ncbi.nlm.nih.gov/tools/primer-blast/). The specificity of primers was validated by melting curve analysis and PCR product sequencing. qRT-PCR was performed on an Applied Biosystems StepOnePlus™ (Thermo Fisher Scientific, Waltham, MA) with iTaq™ Universal SYBR Green Supermix (BioRad, Hercules, CA) using the following conditions for all genes: 95°C for 10 minutes followed 40 cycles at 95°C for 15 seconds, annealing temperature for 20 seconds, and extending at 72°C for one minute.

The geometric mean of mRNA expression of glyceraldehyde-3-phosphate dehydrogenase (*GAPDH*) and actin beta (*β-actin*) has been used as housekeeping genes confirmed by their consistent Ct values among the treatments (*P* > 0.1). Details of primer sequences used for the experiment are presented in Table 2. mRNA expression levels of early markers of adipocyte differentiation, such as peroxisome proliferator-activated receptor gamma (*PPARγ*), fatty acid synthase (*FASN*), CCAAT/enhancer-binding protein alpha (*C/EBPα*), CCAAT/enhancer-binding protein beta (*C/EBPβ*), fatty acid binding protein 4 (*FABP4*), and sterol regulatory element-binding transcription factor 1 (*SREBP1*) were measured, while mRNA expression of secreted phosphoprotein 1 (Osteopontin; *SPP1*), bone morphogenetic protein 2 (*BMP2*), and bone gamma-carboxyglutamic acid-containing protein (Osteocalcin; *BGLAP*) were used to evaluate bone metabolism in marrow tissue. Samples were run in triplicate, and relative gene expression data were analyzed using the 2^-ΔΔCt^. The mean ΔCt of each marker gene from the SO group was used to calculate the ΔΔCt value, and *2*^*-ΔΔCt*^ expression levels were normalized to 1 for the SO group and the FO group expression level presented as fold change.

**Table 2:** Nucleotide sequences of the primers used for quantitative real-time RT-PCR.

Gene^1^	Primer sequence (5’-3’)	Product length (bp)	Annealing temperature (°C)	Accession #
**GAPDH**	F-GCTAAGGCTGTGGGGAAAGT R-TCAGCAGCAGCCTTCACTAC	161	55	NM_204305.1
**β-actin**	F-CAACACAGTGCTGTCTGGTGGTA R-ATCGTACTCCTGCTTGCTGATCC	205	61	NM_205518.1
**C/EBPα**	F-CCTACGGCTACAGAGAGGCT R-GAAATCGAAATCCCCGGCCA	206	60	NM_001031459.1
**C/EBPβ**	F-CCGCTCCATGACCGAACTTA R-GCCGCTGCCTTTATAGTCCT	205	60	NM_205253.2
**PPARγ**	F-GAGCCCAAGTTTGAGTTTGC R-TCTTCAATGGGCTTCACATTT	131	58	XM_025154400.1
**FASN**	F-AGAGGCTTTGAAGCTCGGAC R-GGTGCCTGAATACTTGGGCT	127	60	NM_205155.3
**FABP4**	F-GCAGAAGTGGGATGGCAAAG R-GTTCGCCTTCGGATCAGTCC	153	60	NM_204290.1
**SREBP1**	F-TTCTCAGGGCTGTTCGATGC R-AACACATTGCCGGTAGGGGG	119	60	XM_046927256.1
**BGLAP**	F-GGATGCTCGCAGTGCTAAAG R-CTCACACACCTCTCGTTGGG	142	57	NM_205387.3
**SPP1**	F-GCCCAACATCAGAGCGTAGA R-ACGGGTGACCTCGTTGTTTT	204	57	NM_204535.4
**BMP2**	F-TCAGCTCAGGCCGTTGTTAG R-GTCATTCCACCCCACGTCAT	163	57	XM_025148488.1

^1^GAPDH: **glyceraldehyde-3-phosphate dehydrogenase; β-actin**: actin beta; **PPARγ**: **peroxisome proliferator-activated receptor gamma**; FASN: **fatty acid synthase**; **C/EBPα:** CCAAT/enhancer-binding protein alpha; **C/EBPβ**: CCAAT/enhancer-binding protein beta; FABP4: **fatty acid binding protein 4**; SREBP1: **sterol regulatory element-binding transcription factor 1**; **SPP1**: **secreted phosphoprotein**, **osteopontin**; BMP2: **bone morphogenetic protein 2**; **BGLAP**: **bone gamma-carboxyglutamic acid-containing protein (osteocalcin)**.

### Statistical analysis

All experimental data were expressed as means with standard error of the means (SEM). The differences among the maternal treatment groups were analyzed by one-way ANOVA, whereas the means were analyzed statistically by student’s *t*-test using JMP Pro14 (SAS Institute, Inc., Cary, NC). *p* ≤ 0.05 was considered statistically significant.

## Results

### Maternal dietary EPA and DHA supplementation for breeder hens improve growth performance of offspring at market age by increasing lean mass and total tissue mass

The mortality rate in this experiment was less than 1.0% and was not related to dietary treatments. At the beginning of the experiment, 1-day-old chicks from the FO group had 1.1% lower body weight compared with the SO group (*p* < 0.001) (Table 3). There were no statistically significant differences in body weight gain (BWG) and feed intake (FI) between the two groups at the starter and grower stages. At the finisher stage, BWG and FI increased by 11.57% (*p* < 0.05) and 4.8% (*p* < 0.01) in the FO group, respectively, when compared with the SO group. For the overall period, maternal fish oil supplementation increased BWG by 4.8% (*p* < 0.05) at market age, but it did not affect feed conversion ratio (*p >* 0.05) at any period of the experiment (Table 3).

**Table 3:** Growth performance.

Growth performance	Age^1^	SO	FO	SEM	*p*-value
**Body weight (g)**	day 1	42.7	42.2	0.3	<0.001*
	day 14	374.7	360.9	4.7	0.150
	day 28	1270.8	1274.1	10.5	0.887
	day 42	2145.4	2249.9	26.0	0.018*
**Body weight gain (g)**	Starter	332.0	318.6	4.7	0.180
	Grower	896.1	913.2	7.9	0.303
	Finisher	874.6	975.8	29.1	0.049*
	Overall	2102.7	2207.7	26.0	0.019*
**Feed intake (g)**	Starter	546.5	537.6	10.2	0.684
	Grower	1511.1	1567.4	35.6	0.467
	Finisher	2243.2	2351.8	24.2	0.008*
	Overall	4300.8	4456.8	49.2	0.119
**Feed conversion ratio (Feed intake/Body weight gain)**	Starter	1.608	1.677	0.054	0.276
	Grower	1.688	1.718	0.041	0.370
	Finisher	2.616	2.412	0.098	0.829
	Overall	2.048	2.020	0.029	0.678

^1^ Starter, 1–14 days; Grower, 15–28 days; Finisher, 29–42 days; Overall, 1–42 days. SO, soybean oil group; FO, fish oil group.

* a significantly difference between treatments by using student’s ***t***-test, *p* < **0.05**, N = 6.

Body composition analysis by DEXA indicated that offspring chicks from the FO group had significantly higher body fat percent in 1-day-old chicks (Table 4), where the FO group had a 10.08% higher bone surface area (*p* < 0.05), a 6.85% higher fat mass (*p* < 0.05), and 6.90% higher fat ratio (*p* < 0.01) coupled with a 4.08% lower lean mass when compared with the SO group (*p* < 0.01). There was no difference in total body mass between two groups at 1 day of age. Conversely, at 42 days of age, broilers from the FO group had a 6.70% higher lean mass (*p* < 0.05), and an 11.02% higher total tissue mass (*p* < 0.05) that coupled with a 4.76% higher total body mass (*p* < 0.05) compared to the SO group, but neither fat mass nor bone parameters differed significantly between the groups (*p* > 0.05; Table 4).

**Table 4:** Body composition of broiler chick at 1 day and 42 days of age.

		BMD^1^	BMC	Area	Fat percent	Lean percent	Total tissue	Fat mass	Lean mass
		(g/cm^2^)	(g)	(cm^2^)	(%)	(%)	(g)	(g)	(g)
	SO	0.056	0.68	11.9	36.35	61.84	36.33	13.15	23.04
**Day1**	FO	0.055	0.72	13.1	38.84	56.67	35.88	13.93	22.00
	SEM	0.00004	0.0177	0.3441	0.5022	0.8411	0.2600	0.2100	0.2300
	*p*-value	0.318	0.864	0.041*	0.005*	< 0.001	0.197	0.032*	0.009*
	SO	0.192	41.13	213.83	19.90	80.10	2506.52	500.92	2005.33
**Day 42**	FO	0.192	42.09	219.41	19.60	80.39	2662.13	522.35	2139.75
	SEM	0.001	0.687	2.891	0.327	0.3181	44.40	13.28	34.59
	*p*-value	0.968	0.492	0.345	0.563	0.335	0.035*	0.430	0.021*

^1^BMD, bone mineral density; BMC, bone mineral content; Area, bone area; Fat percent (%), fat percentage; Lean percent (%), lean percentage; Total tissue (g), lean mass plus and fat mass; Fat (g), fat mass; Lean (g), muscle mass; BM(g), total body mass. SO, soybean oil group; FO, fish oil group.

* a significantly difference between treatments by using student’s ***t***-test, *p <* **0.05**, N (Day 1) = 20; N (Day 42) = 18.

### Maternal fish oil altered microstructure of offspring broiler long bone during embryonic development and at market age

**Fig 1:**
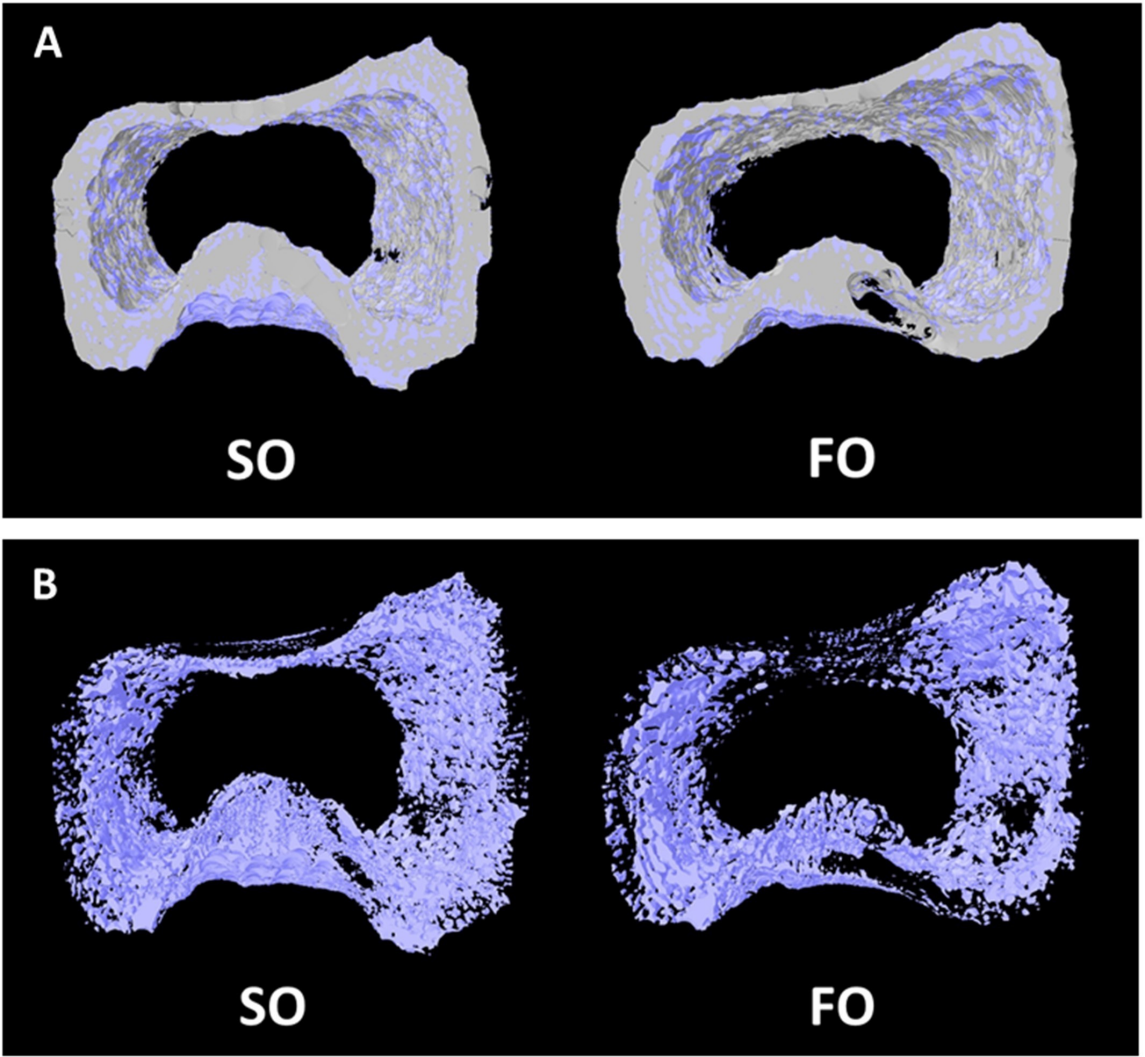
Representative reconstructed 2D images of market-age-broiler femur metaphysis (42 days of age). (A) reconstructed images of cortical porosity (purple) within the cortical bone; (B) lateral view of the reconstructed cortical porosity extracted from bone, indicated the cortical bone pore size, pore volume and porosity ratio.

Femurs of market-age broilers were analyzed by μCT (Fig 1 and Table 5). For total femur bone structure assays, there was a significant decrease of BS/TV in the FO group (*p* < 0.05), whereas there were no statistically significant differences in BMD, BMC, TV, BV, BS and BV/TV between two groups (Table 5). Trabecular and cortical bones of the metaphysis were analyzed after 2-D reconstruction and separating. With maternal fish oil consumption, a numerically lower BMD (*p* = 0.057) was detected in metaphysis trabecular bone, whereas a numerically higher BMD (*p* = 0.054) was observed in cortical bone. Quantitative analysis evidenced a net reduction in the number of open pores (*p* < 0.05) and an increasing number of closed pores (*p* < 0.05) in the FO group. Meanwhile, a smaller pore volume (*p* < 0.05) and a lower total number of pores (*p* < 0.05) were observed in metaphysis cortical bones from the FO group broilers, but no significant change of TV, BV, or BMC were observed.

**Table 5:** Femur metaphysis section 3D structure data at market-age broilers.

	Parameters^1^	Unit	SO	FO	SEM	*p*-value
	**BMC**	g	238.696	234.575	3.74	0.294
	**BMD**	g/mm^2^	0.267	0.263	0.004	0.614
	**TV**	mm^3^	896.422	898.832	14.882	0.531
**Total**	**BV**	mm^3^	338.812	328.551	5.749	0.188
	**BS**	mm^2^	2975.650	2749.910	91.264	0.109
	**BV/TV**	1mm	37.804	36.781	0.508	0.162
	**BS/TV**	1mm	3.304	3.057	0.067	0.044*
	**BMC**	g	1.125	1.140	0.017	0.670
	**BMD**	g/mm^2^	0.115	0.107	0.002	0.057
	**TV**	mm^3^	546.579	565.075	12.085	0.770
	**BV**	mm^3^	56.513	54.026	1.984	0.269
**Trabecular**	**BS**	mm^2^	1287.53	1215.94	46.347	0.224
	**BV/TV**	1/mm	10.328	9.578	0.286	0.096
	**BS/BV**	1/mm	22.743	22.612	0.232	0.392
	**Tb.N**	1/mm	0.645	0.588	0.019	0.067
	**Tb.Th**	mm	0.161	0.163	0.002	0.708
	**Tb.Sp**	mm	1.802	1.954	0.018	0.075
	**SMI**	-	1.673	1.697	0.018	0.749
	**BMC**	g	172.241	168.017	2.920	0.238
	**BMD**	g/mm^2^	0.521	0.539	0.006	0.054
	**TV**	mm^3^	332.857	312.124	6.959	0.068
	**BV**	mm^3^	278.233	267.363	5.122	0.145
**Cortical**	**Po.N(cl)**	-	83.809	85.733	0.509	0.028*
	**Po.V(cl)**	mm^3^	1.318	1.407	0.074	0.723
	**Po. S(cl)**	mm^2^	66.732	68.688	3.552	0.607
	**Po (cl)**	%	0.460	0.523	0.022	0.916
	**Po (op)**	%	15.803	13.819	0.514	0.025*
	**Po.V(op)**	mm^3^	53.306	43.353	2.507	0.022*
	**Po.V(tot)**	mm^3^	54.623	44.760	2.527	0.024*
	**Po (tot)**	%	16.190	14.271	0.509	0.028*

^1^BMC, bone mineral content; BMD, bone mineral density; TV, total bone volume; BV, bone volume (TV minus bone marrow volume); BS, bone surface area; BV/TV, bone volume/total volume; BS/TV, bone surface/total volume; Tb. N, trabecular number; Tb. Th, trabecular bone thickness; Tb. Sp, trabecular spacing; SMI, structural model index; Po. V(tot), total volume of pore space; Po (tot)%, percentage of pores; Po. N(cl), number of close pore; Po. V(cl), volume of close pore; Po. (cl), close porosity (percent); Po. S(cl), close pore surface; Po. V(op), volume of open pore; Po. (op), open porosity (percent). SO, soybean oil group; FO, fish oil group.

* a significantly difference between treatments by using student’s ***t***-test, *p* < 0.05, N = 18.

**Fig 2:**
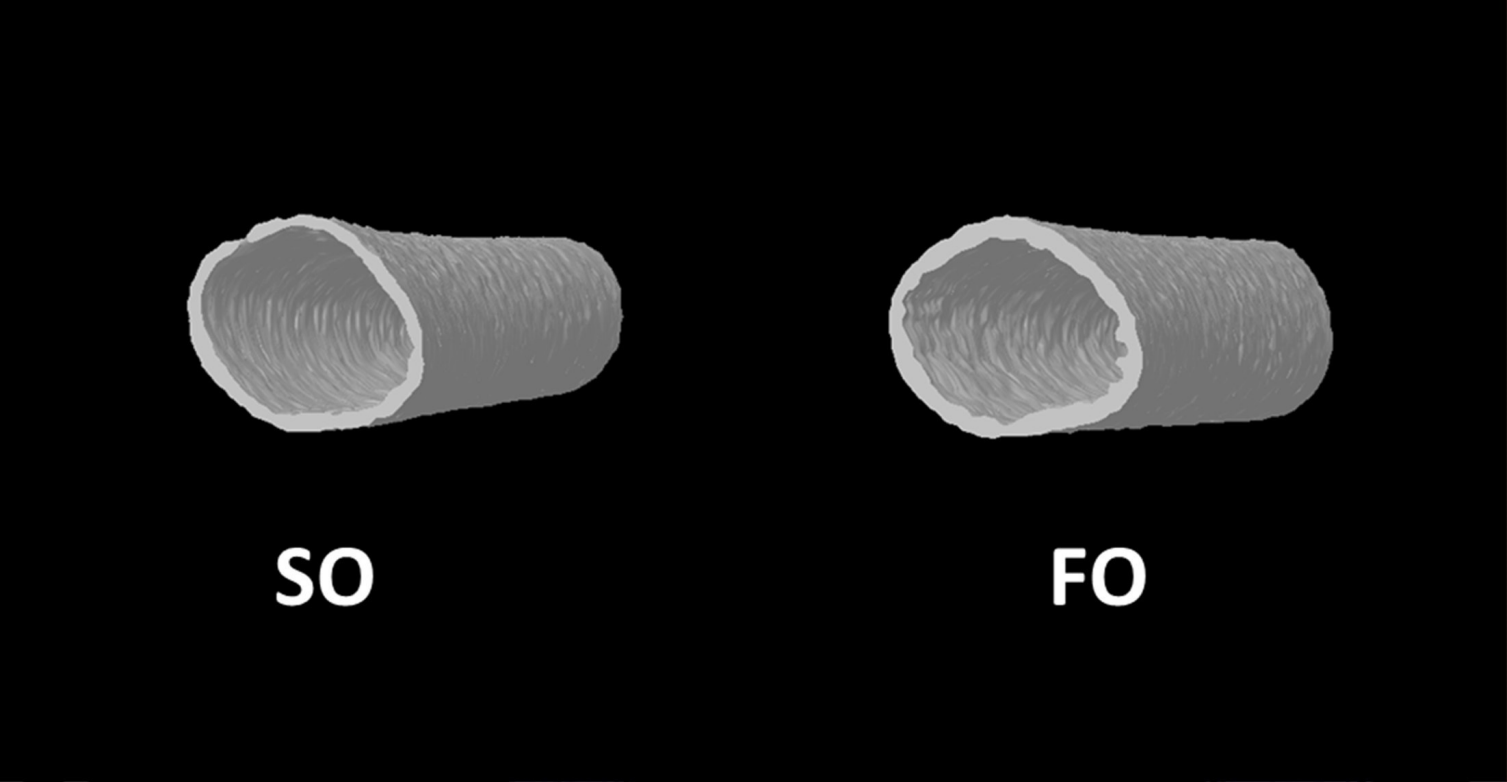
Reconstructed 2D structure of tibia diaphysis on day 18 embryo. Haversian artery was used as a landmark for region of interest (ROI) selection. The diaphysis bone traits were scanned and analyzed by μCT.

Based on these findings, we went back to evaluate early effects of maternal fish oil on embryonic bone development (Fig 2). Maternal fish oil supplement significantly increased BMC (*p* < 0.01), TV (*p* < 0.05), and BS (*p* < 0.05) and decreased porosity volume (Po. V(tot)) (*p* < 0.05) on tibia of 18 days old embryo. However, there were no statistically significant differences in BMD, BV, TS or percentage of pores on embryonic bone between two groups (Fig 2 and Table 6).

**Table 6:** Metaphysis microstructure of tibia on day 18 embryo.

	Unit	SO	FO	SEM	*p*-value
**BMC** ^**1**^	g	4.007	4.520	0.139	0.003*
**BMD**	g/mm^2^	0.269	0.278	0.004	0.155
**TV**	mm^3^	14.561	16.225	0.427	0.024*
**BV**	mm^3^	7.040	7.843	0.249	0.055
**TS**	mm^2^	41.011	42.741	0.632	0.089
**BS**	mm^2^	61.248	65.887	1.046	0.011*
**Po.V(tot)**	mm^3^	8.382	7.522	0.221	0.002*
**Po (tot)**	**%**	**51.820**	**51.681**	**0.719**	0.537

^1^BMC, bone mineral content; BMD, bone mineral density; TV, total bone volume; BV, bone volume (TV minus bone marrow volume); TS, total surface area; BS, bone surface area; Po.V(tot), total volume of pore space; Po (tot)%, percentage of pores.

* a significantly difference between treatments by using student’s ***t***-test, *p* < 0.05, N = 10.

### Maternal fish oil treatment suppressed adipogenic gene expression in offspring broiler bone marrow tissue

**Fig 3:**
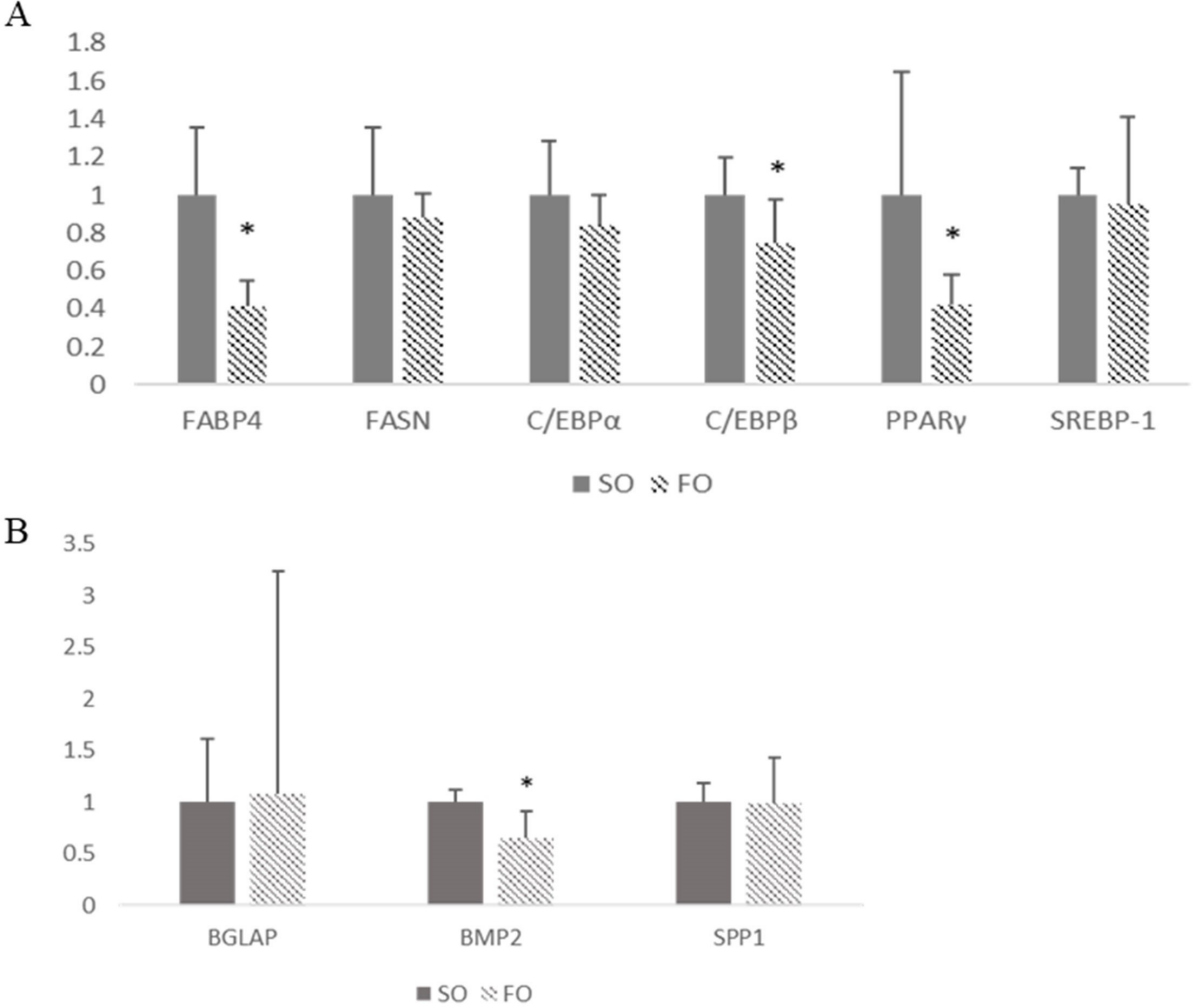
The relative mRNA expression of adipogenesis and osteogenesis marker genes. Maternal fish oil supplementation suppressed bone marrow adipogenesis but didn’t change osteogenesis in offspring broiler. Expression of A) adipogenesis marker genes; B) osteogenesis marker genes; The relative mRNA expressions were detected by qPCR method in SO and FO group. Total RNA from femur bone marrow from 42 day-old-broiler were collect and reversed (N = 6). Fold changes of gene expression were calculated using the ΔΔCt method by student’s *t*-test. Each experiment was repeated at least 3 times. The error bars represent SEM. * means there was a statistically significant difference between treatments, *p* < 0.05.

Expression of genes involved in adipogenesis and osteogenesis in femur bone marrow tissue of the SO and FO broilers at 42 days of age. Potential mechanisms for the difference in adiposity were evaluated based on expression of genes that mediate lipogenesis and adipogenesis. As shown in Fig 3A, significant downregulation of *PPARγ*, *FABP4*, and *C/EBPβ* was found in the FO group marrow tissue compared with the SO group (*p* < 0.05). However, there was no difference between the two groups for expression of *FASN*, *SREBP1*, or *C/EBPα*.

Potential mechanisms for the difference in bone metabolism and growth were evaluated based on expression of genes that mediate osteogenesis and bone formation. *BGLAP* and *SPP1* did not differ significantly between the groups (Fig 3B; *p* > 0.05), whereas the expression of *BMP2* was downregulated in the FO group (*p* < 0.05).

## Discussion

Both omega-3 and omega-6 fatty acids are essential for health and need to be consumed in proper balance [48, 49]. In the present study, maternal fish oil diet significantly improved the growth performance in offspring broilers at market age when compared with soybean oil. Increased BW was primarily due to an improvement in lean muscle gain. Based on the present results, we concluded that the prenatal effects of fish oil from the hen diet improved bone quality and increased lean mass of offspring broilers at market age. In the current study, femur microstructure of market age broilers was analyzed by μCT. Femur is one of the most mineralized bones in the skeletal system and also a good indicator of overall skeletal mineralization microstructural properties [50]. Most studies use total bone BMD or BMC as a common parameter to evaluate bone quality, but morphometry and biomechanical analyses also indicate that impaired cortical bone strength is also a consequence of increased porosity [51, 52], and greater porosity is associated with higher odds for bone fracture [53–56]. Therefore, porosity is a substantial determinant of the bone fragility and mechanical competence and can be a target for bone abnormalities prevention in broilers [50, 57]. In the present study, although μCT and DEXA showed that there were no significant differences in BMD or BMC between the two groups, but femur microstructure study illustrated a numeric increase of BMD in central diaphysis cortical bone area as well as a lower micropore volume that coupled with higher closed pore ratio, indicating a positive impact of maternal fish oil diet on bone quality in offspring broilers at 42 days. Meanwhile, at the market age of broilers, bone pore size and pore number are the key anatomic traits to reflect unbalanced bone homeostasis. As a dynamic organ, the bone structure depends on not only bone formation but also bone resorption. Larger pore size is highly associated with increased cortical remodeling that leads to impairment in elasticity, strength, and toughness of the bone [56]. The major hypothesis for such bone health studies with fish oil was n-3 PUFA could affect stem cells differentiation, cell population, and cell activity in bone marrow to enhance bone formation [58, 59]. The long bones metaphysis contains large amounts of marrow adipose tissue and hematopoietic red marrow, where marrow composition and adipocyte proportion tend to change in response to nutrition and environmental stress [60, 61]. It has been reported that lipid profiles in marrow showed an increase in the proportion of unsaturated fatty acids by fish oil, and dietary fish oils reduced the amount of lipid in bone marrow [62, 63]. The high level of n-3 PUFA intake could directly alter the lipid profile in bone marrow by increasing n-3 PUFA concentration in bone marrow and optimize bone formation by altering the production of bone growth factors [19, 64]. It is also known that bone resorption is accomplished by bone-resorbing cells known as osteoclasts, and the activity and functions of osteoclasts are regulated by several receptor activator such as receptor activator of nuclear factor NF-kB ligand (RANKL). In rat, dietary intake of DHA during early post-weaning could suppresses adipogenesis, enhances bone marrow cell number [65], and introduces stem cells into the osteoblastic lineage by enhancing bone-specific transcription factors [66]. Meanwhile, perinatal or dietary n-3 PUFA supplementation can decrease the number of osteoclast cells *via* not only modulating mRNA expression of RANKL in the rat [67, 68] but also mediating osteoclast activity, inhibiting bone resorption during bone remodeling [23, 68, 69]. The dual effects of n-3 PUFA on both adipose tissue and bone development are due in part to the shared developmental origin of adipocytes and osteoblasts, both of which originate from stem cells during embryonic development [69]. Bone marrow mesenchymal stem cells (MSCs) differentiate into either osteoblasts that contribute to bone density, or adipocytes that comprise the fat fraction of marrow [70]. Bone marrow adipose tissue is a large portion of the bone marrow content and plays an important role in energy storage, endocrine function, and bone metabolism [71–73]. However, excess adipogenesis in bone marrow tissue is adversely correlated with bone quality, causing bone disorders such as osteoporosis [55, 60, 74]. Notably, compared with n-6 PUFA, dietary n-3 PUFA supplementation can down-regulating *PPARγ*, the master regulator of adipogenesis, suppressing adipocyte formation in bone marrow of rodent and broilers [62, 64, 75–78]. Although the previous studies do not assess the effects of fish oil on bone development between between avian and other mammal species, it is reasonable to speculate that developmental programming with LC n-3 PUFA may have similar benefits for bone strength in broilers. The present study showed that the expression of the adipogenic transcription factors, including *PPARγ*, *C/EBPβ*, and *FABP4* were downregulated in the bone marrow of the FO broilers, with a lower bone porosity. This finding is in line with the hypothesis that maternal fish oil supplement has inhibitory effect on adipocyte differentiation of MSCs, that could drive the improvement of bone health in offspring broilers [79–82]. Although the present study found that the FO group had significantly lower *BMP2* which plays an important role in the development of cartilage and bone, several studies have showed that *BMP2* is also expressed in adipose tissue and preadipocytes in human [83], and *BMP2* supports both adipogenic and osteogenic differentiation in various progenitor cells, dependent on treatment, culture condition, and cell types [84–86]. *BMP2* treatment on bone marrow MSCs in adipogenic medium increased *PPARγ* activation [87, 88], indicating the important role of *BMP2* not only in bone metabolism but also in adipogenesis. Based on the present results, the significant suppression of *BMP2* in FO group, therefore, possibly account for the down-regulation of *PPARγ* as the results of decreased adipogenesis in bone marrow. Besides, compared with adipogenesis and osteoclastogenesis, fish oil has relatively mild effects on bone osteoblastogensis, suggesting that osteogenic responses were relatively less sensitive in bone marrow [69, 89]. The bone formation and development can respond to mechanical stress and environment change [90, 91]; thus, with the increasing body weight gain, a higher mechanic stress might have been loaded on femurs of the FO group compared to those of the SO group.

We further evaluated the effects of maternal fish oil diet on embryonic bone traits by using μCT method. Results showed that maternal fish oil supplement significantly improved the bone traits during later embryonic development when compared with soybean oil group, by increasing BMC and BV and decreasing porosity volume. These results coincided with a previous study on Japanese quail, where with maternal fish oil supplementation, thicker cortical bones, higher shear force responses, and higher bone breaking strength were observed in the tibia diaphysis area [18, 20]. As with many previous studies, we concluded that the increasing of n-3 PUFA ratio in hen diet could optimize progeny bone health and growth performance. There are several potential mechanisms related to prenatal n-3 PUFA supplement and embryonic bone health. Maternal dietary supplement of n-3 PUFA can be incorporated in the egg yolk to become available for the developing embryo, which directly modify fetal growth programming and epigenetic regulation [43, 92–94]. The modulation might directly be associated with the expression of bone-related proteins, or differential methylation profiles, contributing to superior physical structure and better bone quality [95]. Besides, dietary fish oil supplementation could increase calcium absorption in the small intestine and improve bone mineralization and quality in laying hens [15, 96, 97]. In addition, fish oil enriched diet also improves reproductive performance, organs functionality, immunocompetence, skeletal health, and gastrointestinal development in breeder hens [14, 16, 93].

Considering the management and cost in large production, fish oil may not be the most practical option [98]. Therefore, a replacement of fish oil from other sources would be another option. Recent studies have reported that fish byproducts [99], microalgae [100], and flaxseed oil [101] are more cost-effective and have been applied in poultry production. Thus, those products have potential to be used for developmental programming through the hen diet. Besides, in comparison to mammals, avian species have relatively enhanced ability to synthesize long chain n-3 PUFA species from their alpha-linolenic acid (ALA; 18:3 n-3) precursor due to the unique properties of avian elongases enzymes [102–105]. Plant-based ALA feeding significantly reduced the cholesterol and fat percentages of meat in broilers [106, 107]. However, with the inconsistency in the literature, some studies finding no effects or negative effects of n-3 PUFA inclusion on bone quality in avian or the other species [67, 108–110]. We hypothesize that these variations can likely due to the source and quality of n-3 PUFA. Furthermore, the impact of n-3 PUFA supplementation depends on the levels of EPA and DHA [42, 111]. Studies pointed out that health benefits of n-3 PUFA are heavily depending on the source, dose, and duration of n-3 PUFA enrich diets [105, 112–114]. For example, ALA as source of n-3 PUFA, the conversion ratio of ALA to DHA/EPA might not be efficient to improve growth with a short period of treatment [115]. Compared with other animal models, broilers have very short life spans, and the tissue growth is extremely efficient, considering the weight gain and muscle growth. Thus, the potential beneficial effects of LC n-3 PUFA consumption on bone health could be limited by the treatment time. Thus, further studies are necessary to maximize the beneficial effects of LC n-3 PUFA.

In conclusion, our observations demonstrated that maternal fish oil diet rich in n-3 PUFA could have a favorable impact on fat mass and skeletal integrity in broiler offspring. Our findings provide a novel nutrition strategy using maternal fish oil to prevent bone disorders and improve meat yield in offspring broilers.

## Supporting information

S1 Data (DOCX)
