# Supplementary material for: The effects of maternal fish oil supplementation rich in n-3 PUFA on offspring-broiler growth performance, body composition and bone microstructure
Source: PLoS One. 2022 Aug 16;17(8):e0273025. doi: 10.1371/journal.pone.0273025 (PMC9380956; doi:10.1371/journal.pone.0273025)
Supplement: S1 Data — (DOCX) [file pone.0273025.s001.docx]

Website: Figshare

Minimal data set

Data location: 10.6084/m9.figshare.20301495

- The values used to build graphs;

Figshare DOI:

10.6084/m9.figshare.20301870
